# Opinions Differ

**Published:** 1888-08

**Authors:** 


### OPINIONS DIFFER.

In these days of Talmagian ranters and namby-pamby ceremonialists, it is refreshing to turn to a common sense discourse like the following, delivered by the late Henry Ward Beecher, from Plymouth pulpit, September 27, 1855, an epetometized report of which appeared in the New York *Herald* the day following, in these words :

"The Personality of God" was the subject upon which Mr. Beecher preached in the morning at Plymouth Church. "The human mind cannot well conceive of divine personality," he said. "There is nothing like it among men, although there are intimations of it. Among men personality involves form, but God is formless, and almost from the very beginning forbade man to liken him to anything. The idea of personality in God is absolutely unthinkable ; and yet we believe in the personality of God ; that He is a being such as no other being is ; that in His own way and sphere and spiritual kingdom He will be discernible.

The Hebrew mind conceived of God as being everywhere present ; the Greek accepted the idea that He was in creation, that all creation stood upon him, as it were—drew life from His bosom. The Roman mind was a hard, but regal mind. It attempted to reduce everything to precision. Roman theology accepted the teaching of the Bible that God was everywhere, but it felt that God was like an engineer who, having made a locomotive, stood off and watched its performances, saying, 'Let her go,' and admired or criticised it as might be. The Roman mind has perpetuated the idea that God is in heaven, that we are on earth, and that, having created the earth and wound it up, He sees to it that the keys are not lost, and forever keeps the engines in play, looking on from above to see what the result may be ; that He is a great magistrate who hears the reports of his police every day, and He sits in the Temple of Justice or the Temple of Love according to the individual conception.

Modern soience believes in God as a great underlying energy. Scientists say that there is no God in fact ; that once postulating energy the whole of science can be unfolded from it. Yes, they call it energy ; I call it God. His is a personality that we can form no conception of, but nevertheless, He is in the world. He is the swaddling clothes, as it were, of the infant, He is the raiment of the man. The prevalent misconception of the universality of God gives a clearer conception to the mission of Jesus Christ. Men have yearned to bring down God just for one moment that they might know Him as He is. They longed to facilitate their intercourse with God. This is what Jesus did: He became like men ; His blood pulsed as theirs ; He became imprisoned in matter, and thus, while reduced to the condition of humanity, He lived in the equality of the Godhead, and man perceived in Him, exactly harmonized, what were the divine disposition and the Divine attributes. Then the world had something that it could fix its eye upon, which answered to its longing for personality.

The old theology says that Christ came into the world to save a lost race. There was, there is, no lost race. It says that He came to make good Adam's stumble ; but Adam never existed, and, consequently, never stumbled. It is time that all this mechanical, ladder-like machine sort of salvation were done away with. This merit and demerit, deserving and non-deserving, is all artificial, all pagan. God saves who will be saved. Whoever recognizes God in him and around him and goes in the direction which He has made manifest that man is saved, not because anything has been done for him in the way of an atonement.

There is many a horse that is more deserving of immortality than the man who rides it. There is many a dog that has more disinterested love than the man who owns it. Why shouldn't they have a chance hereafter ? I don't know but they will. That is something I don't know anything about."

